# Editorial: The oral microbiota in mental health

**DOI:** 10.3389/fpsyt.2022.1048179

**Published:** 2022-10-07

**Authors:** Stefanie Malan-Müller, Teodor T. Postolache

**Affiliations:** ^1^Department of Pharmacology and Toxicology, Faculty of Medicine, Universidad Complutense de Madrid, Madrid, Spain; ^2^Biomedical Network Research Center of Mental Health (CIBERSAM), Institute of Health Carlos III, Madrid, Spain; ^3^University of Maryland School of Medicine, Baltimore, MD, United States; ^4^Military and Veteran Microbiome: Consortium for Research and Education, Aurora, CO, United States; ^5^Rocky Mountain Mental Illness Research Education and Clinical Center, Rocky Mountain Regional Veterans Affairs Medical Center, Aurora, CO, United States

**Keywords:** periodontitis, oral microbiota, oral health, inflammation, microbiome, anxiety, depression, oral-brain axis

The importance of the microbiota-gut-brain axis has been of major interest in mental health research in recent years. In addition, research endeavors have expanded to also include the role of the oral microbiota in mental health. The articles published as part of this Frontiers Research Topic provide insight into the bidirectional connection between periodontal and mental health and the reciprocal interaction between the oral microbiota and central nervous system (CNS). This bidirectional axis provides potential targetable molecular, cellular and behavioral interventions that might contribute to the prevention of precipitation, reactivation, exacerbation, and progression of mental health disorders, to be investigated in controlled experimental and clinical models.

## Epidemiological findings linking oral and mental health

The oral cavity houses the second largest set of bacteria in the body, and its alteration is responsible for the two most prevalent oral diseases: caries and periodontal diseases, as reviewed by Martinez et al. Furthermore, Bowland and Weyrich and Martinez et al. also review epidemiological data on associations between periodontitis and neurodegenerative and neuropsychiatric disorders (NPDs) and discuss immune activation as one of the main driving forces that links these disorders. Periodontitis is a multifactorial, chronic inflammatory disease caused by dysbiosis in the subgingival microbiota and causes low-grade systemic inflammation *via* pro-inflammatory cytokine release and local and systemic invasion by certain periodontal pathogens. What's more, many NPDs are linked to neuroinflammation.

Although both NPDs and periodontal diseases are linked to inflammation, determining the causative relationship between periodontitis and NPDs has proven, difficult, since most studies are cross-sectional. Martinez et al. reviewed data from a longitudinal, large-scale study that suggested that periodontitis could be a risk indicator for the subsequent development of depression; however, evidence is scarce, and NPDs could, in turn, drive inflammation and exacerbate oral diseases.

## The oral-brain axis and beyond

Following epidemiological observations that associate periodontitis and NPDs, with inflammation as a potential link between them, researchers hypothesized that a bidirectional oral-brain axis may be involved and set out to identify functional mechanisms and pathways that link the oral cavity with the CNS. As reviewed in Martinez et al., preclinical studies confirmed that induction of periodontitis resulted in anxiety and depressive-like behaviors as well as immune activation, and functional and chemical changes in the CNS. To disentangle the bi-directional relationship between periodontitis and NPDs, the use of combined animal models—using stress exposure together with periodontitis induction—is fundamental. The combined models reported HPA axis dysregulation together with a hyperinflammatory response, often accompanied by behavioral changes. This provided more concrete evidence for the existence of an oral-brain axis.

Bowland and Weyrich proposed a hypothetical framework for a functional oral-microbiota-brain axis (OMBA), which includes microbial and metabolite escape, neuroinflammation, CNS signaling, and response to neurohormones, and they elaborated on how the OMBA connects microbial dysbiosis and NPD progression and outcome. Martinez et al. expanded this axis to also include the gut microbiota in their proposed multidirectional oral-gut-brain axis. Periodontitis can result in a leaky periodontium and bacteria, lipopolysaccharides or immune mediators can reach systemic circulation (hematogenously), which can activate the HPA axis and result in increased stress hormones, which in turn influence gut physiology and gut microbiota. An altered gut microbiota can result in systemic inflammation, which affects the CNS and exacerbates other inflammatory pathologies. Periodontal bacteria can directly influence the gut microbiota *via* enteral transmission or indirectly *via* hematogenous transmission. Both papers by Bowland and Weyrich and Martinez et al. elaborate on different parts of the oral-microbiota-brain axis and Bowland and Weyrich provide an interesting anthropological perspective on how sociocultural and environmental factors influence the oral microbiota and mental health and provide future directions for this line of research.

Clinical studies on the oral microbiota in the context of mental health outcomes are limited. Furthermore, in NPD patients, particular behaviors, lifestyle, or disease-associated factors [phobias or fears toward oral health interventions, higher levels of cigarette smoking in patients and dry mouth (xerostomia) caused by psychotropic medications] can affect the oral microbiota composition and should be considered carefully when performing these studies. Al Bataineh et al. investigated the link between smoking, the oral microbiota, and avoidance behavior, using the behavioral activation for depression scale (BADS). The abundance of the phyla Bacteroidota, Campylobacterota, Firmicutes A, Firmicutes I, and Fusobacteriota were significantly increased in the avoidance group, whereas Verrucomicrobiota was significantly lower in the avoidance risk group. These findings correlate with previous oral microbiome and mental health data: the *Prevotella* genus (phylum Bacteroidota) has been associated with diseased periodontal tissue, inflammation, and measures of distress (reviewed by Martinez et al.), whereas *Fusobacterium nucleatum* and *Leptotrichia* (phylum Fusobacteriota) were correlated with diseased periodontal tissues and periodontal disease, respectively, and were positively correlated with depression (reviewed by Martinez et al.).

## The oral-brain axis as a potential therapeutic target

Since periodontal diseases may play a role in certain systemic diseases, such as diabetes and cardiovascular disease, it is vital to maintain periodontal health to promote general health and wellbeing. Although no research is currently available, to our knowledge, to illustrate that periodontal interventions could improve mental health outcomes, treatment of periodontitis promotes oral health by decreasing local and systemic inflammation. Martinez et al. discuss several oral microbiota-targeting treatments used adjunctly to mechanical disruption of subgingival biofilm, and how these could potentially be used to improve mental health symptoms. Lastly, Bowland and Weyrich also reviewed approaches that target the oral microbiota to treat NPDs and emphasized the importance of the One Health perspective, where our health is reliant on the external health of our environments and ecosystems. They proposed that researchers should determine how exposure to environmental microbes may influence oral health and suggest that a One Health approach could inform health policies aimed at targeting oral and mental health.

Together, these articles suggest a role for the oral microbiota in mental health within the oral-brain axis and propose the oral microbiota as a novel therapeutic target to improve mental health outcomes.

## Author contributions

SM-M: elaboration, conceptualization, basic and clinical comments on the manuscript. Both authors contributed to the article and approved the submitted version.

## Funding

SM-M is supported by UNA4CAREER H2020-Marie Skłodowska-Curie Actions co-fund research grant (UNA Europa, an alliance of Universities FOR the Emergence of Talent and the Development of Research CAREERs; Grant number 847635). TP's work is supported, in part, by the Rocky Mountain MIRECC, Aurora Colorado, USA and the Military and Veteran Microbiome Consortium for Research and Education (MVM-CORE), Aurora, Colorado, USA and by the Center for Sleep, Mood, Anxiety and Depression, Washington, DC, USA.

## Conflict of interest

The authors declare that the research was conducted in the absence of any commercial or financial relationships that could be construed as a potential conflict of interest.

## Publisher's note

All claims expressed in this article are solely those of the authors and do not necessarily represent those of their affiliated organizations, or those of the publisher, the editors and the reviewers. Any product that may be evaluated in this article, or claim that may be made by its manufacturer, is not guaranteed or endorsed by the publisher.

## Author disclaimer

The editorial represents the opinion of the authors and not the official position or statements of the US Veterans Health Administration.

